# IgG4-Related Disease With Renal and Pulmonary Involvement

**DOI:** 10.7759/cureus.17071

**Published:** 2021-08-10

**Authors:** Chinenye Osuorji, Kiron Master, Ikenna Osuorji

**Affiliations:** 1 Internal Medicine, Burrell College of Osteopathic Medicine, Las Cruces, USA; 2 Radiology, Hospitals of Providence Sierra Campus, El Paso, USA; 3 Hematology and Oncology, Burrell College of Osteopathic Medicine, Las Cruces, USA

**Keywords:** igg4 -related disease, storiform fibrosis, lung nodules, tubulointerstitial fibrosis, plasma cells

## Abstract

Immunoglobulin G4-related disease (IgG4-RD) is a rare immune-mediated disease affecting multiple organs and tissues. There is often the presence of elevated serum Ig4 subtype with histological evidence of lymphoplasmacytic infiltration, fibrosis, and phlebitis. The mainstay of treatment is steroids therapy. We report the case of a 66-year-old man presenting with acute on chronic renal failure and pulmonary nodules seen on PET-CT scan. He also had elevated serum IgG4 subclass and histological features in keeping with IgG4-RD. He failed steroid therapy but responded subsequently to rituximab with complete resolution of his symptoms.

## Introduction

IgG4-related disease (IgG4-RD) is a chronic systemic inflammatory disorder typically characterized by extensive infiltration of organs by lymphoplasmacytic cells, presence of storiform fibrosis, and obliterative phlebitis. It remains a diagnostic challenge as it is often mischaracterized as a malignant disease especially due to its tumor-like presentation and multi-organ involvement [1].

## Case presentation

A 66-year-old Hispanic man presented with a rapid decline in glomerular filtration rate (GFR) of about 50% from baseline in a 2-year period. Initial evaluation revealed elevated serum IgG4 subclass levels of 1340 (normal range: 4-86 mg/dl). He denied any other urinary symptoms; no weight loss or constitutional symptoms was reported. At baseline, he had a history of stage 3 chronic kidney disease secondary to hypertensive nephrosclerosis, essential hypertension, well-controlled type 2 diabetes, asthma, and prior corrective surgery for a deviated nasal septum. His colonoscopy report was up-to-date and unremarkable. Physical examination and review of systems were unremarkable. 

**Figure 1:**
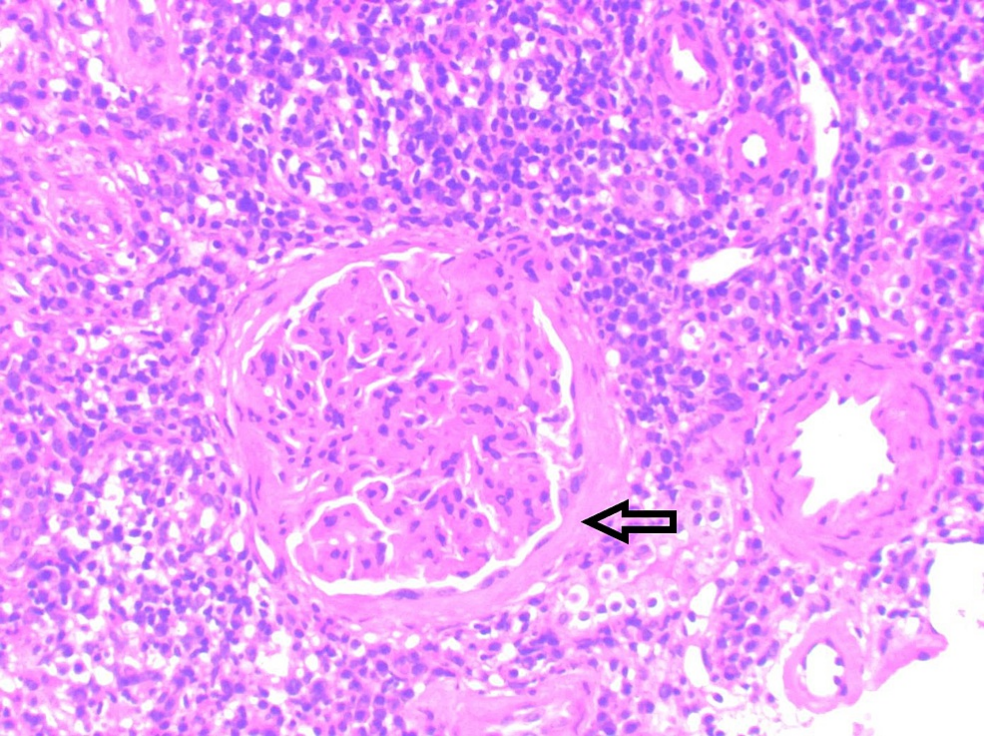
(10X) H&E microscopic images showing glomeruli displaying periconcentric storiform fibrosis and generalized mesangial hypercellularity surrounded by marked lymphoplasmocytic inflammation

**Figure 2:**
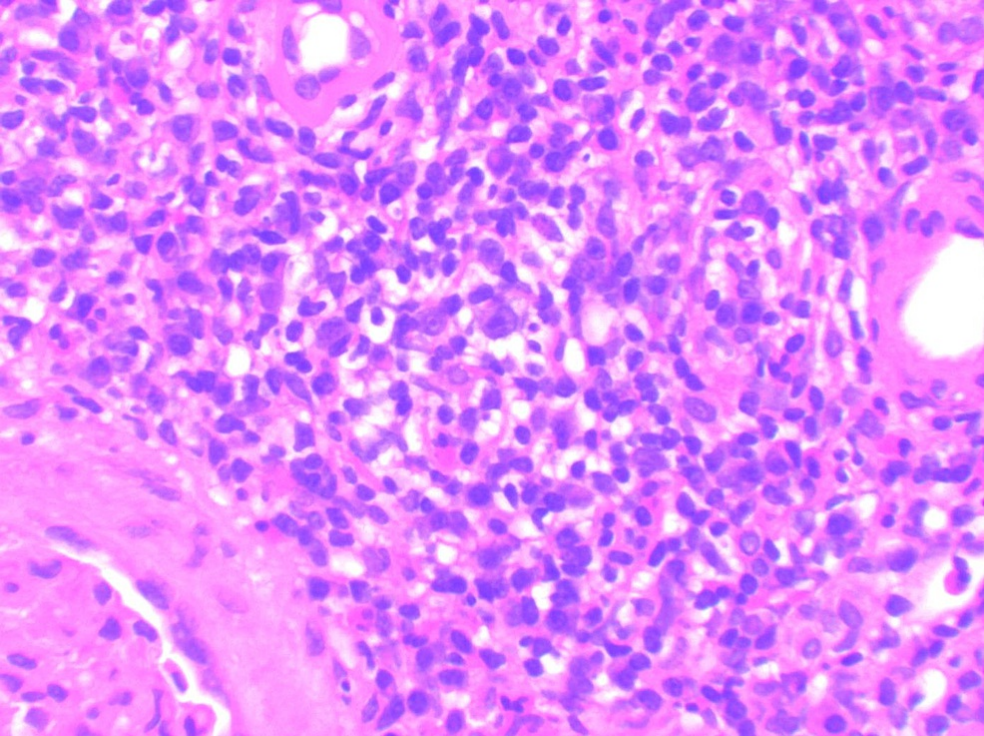
(20X) H&E microscopic images showing lymphoplasmocytic interstitial inflammation

The patient’s complete blood count result showed a white cell count of 5200 cells/dl with normal differentials. Hemoglobin was 14.4g/dl. He had normal liver enzymes. Serum IgG4 level of 1340 mg/dl, creatinine of 1.5, BUN of 23, EGFR of 45, glucose of 145, and hemoglobin A1c of 6.7. ANCA titers were not obtained. Urine analysis revealed trace blood and trace leukocyte esterase, but was otherwise unremarkable. Renal ultrasound revealed normal-sized kidneys of 12cm and 12.1cm on the right and left, respectively. Urine and serum Immunoelectrophoresis showed no monoclonal immunoglobulins or monoclonal free light chains. The patient had a left kidney biopsy that showed features of chronic interstitial nephritis with increased IgG4 positive plasma cells (Figure 1, 2). 

**Figure 3:**
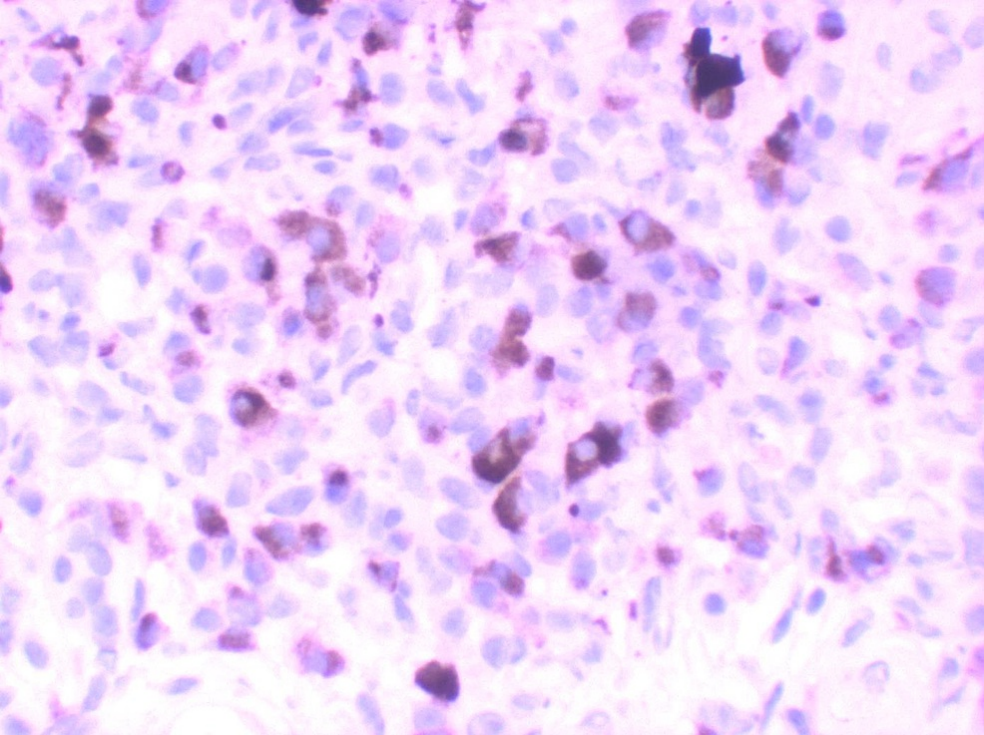
IgG4 Immunohistochemical stains displaying the presence of increased numbers of positive plasma cells (40X)

**Figure 4:**
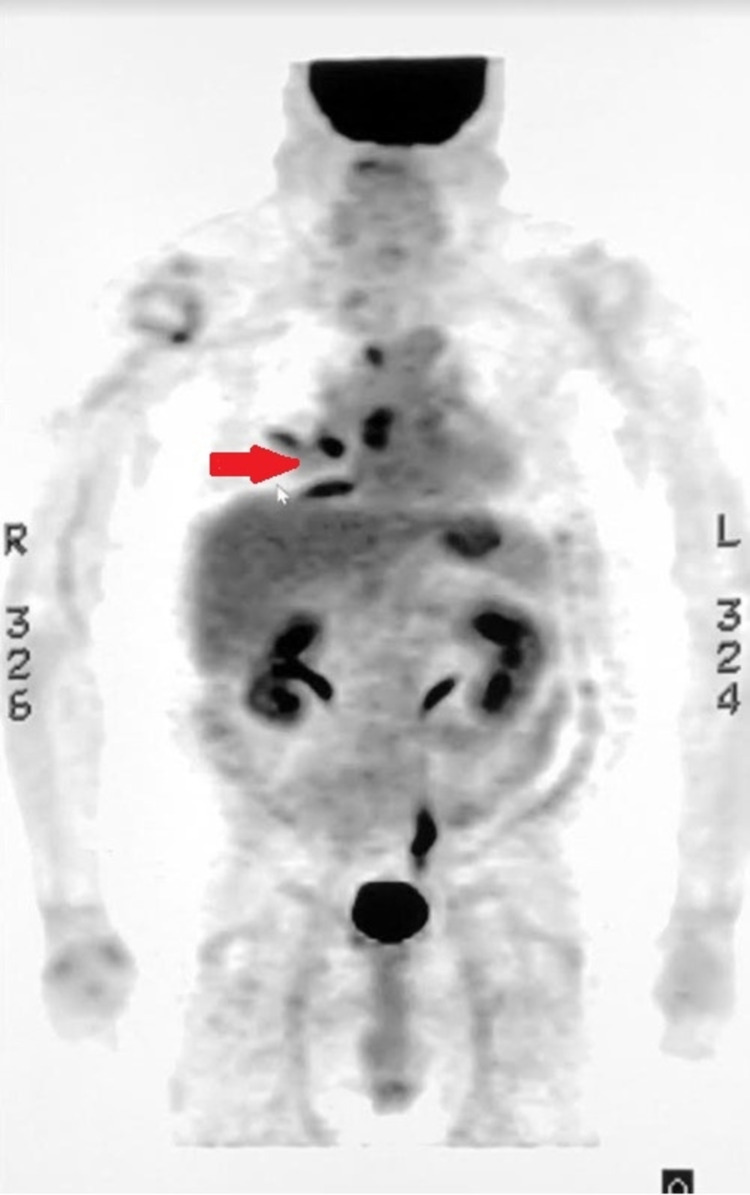
PET/CT scan which showed a large FDG avid right hilar and mediastinal lymph nodes with right paratracheal and subcarinal distribution, dense right lung middle lobe consolidation with mild medial right lung measured 1.9 X1.4 cm with SUV of 5.9

In the interstitial compartment, there was the presence of lymphoplasmacytic infiltrates that approximately compromised 60% of the renal parenchyma. The plasma cells were IgG4 positive (focally displacing and IgG4 to IgG ratio of greater than 0.4 with greater than 10 IgG4-positive plasma cells per high power field (Figure 3). There was no evidence of immune complex-mediated glomerulonephritis, hereditary nephritis, vasculitis or dysproteinemia-related nephropathy. Positron emission tomography (PET/CT) scan showed a large FDG avid right hilar and mediastinal lymph nodes with right paratracheal and subcarinal distribution. The maximum SUV of mediastinal lymph nodes was 5.5 with subcarinal lymph nodes measuring 2.8 X 1.9 cm. PET scan also revealed dense right lung middle lobe consolidation with mild medial right lung measured 1.9 X1.4 cm with SUV of 5.9 (Figure 4).

**Figure 5:**
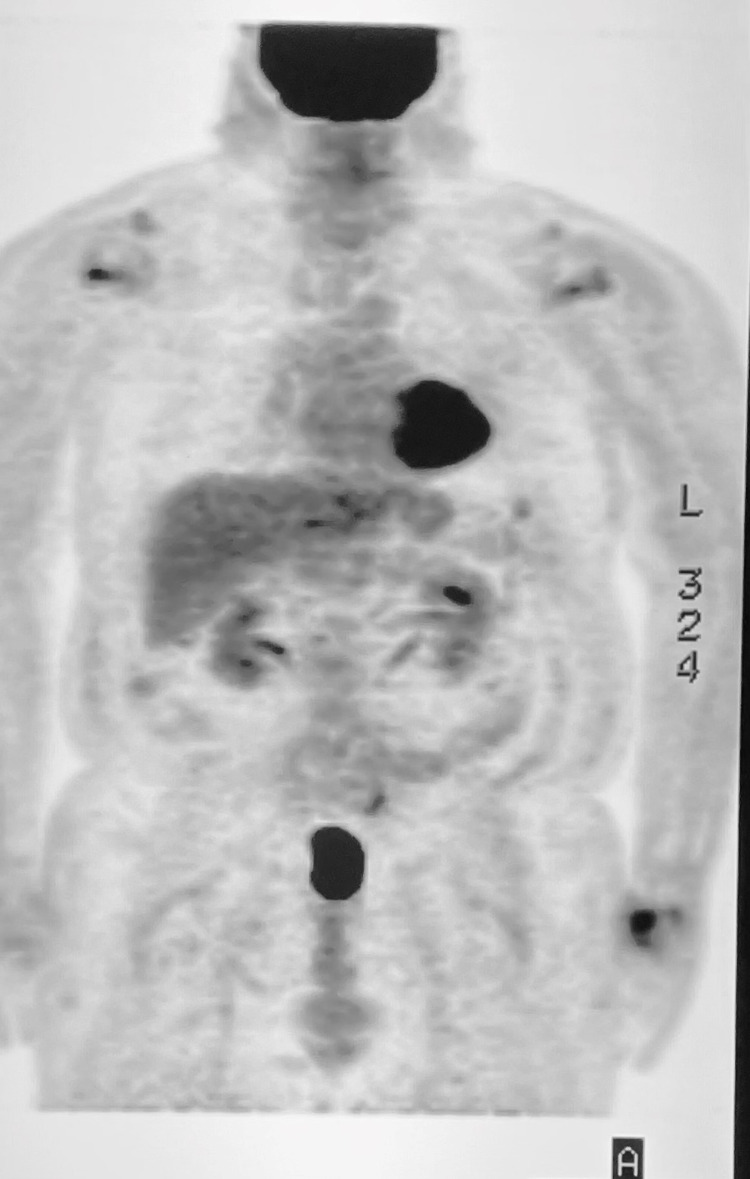
PET-CT six months post treatment with IV rituximab showing complete resolution of lesions.

He was diagnosed and treated for IgG-4 related renal failure and respiratory disease. He had a suboptimal response to initial treatment with oral prednisone, 50mg daily for three months. His renal function continued to decline but improved significantly when IV rituximab was initiated. He received two doses of rituximab IV infusions 1000mg at two weekly intervals, pre-medicated with 25mg diphenhydramine, 100mg IV methylprednisolone, and 650mg acetaminophen. Repeat PET-CT six months later showed interval resolution of pulmonary lesions (Figure 5). His serum creatinine levels also improved and remain stable over the course of treatment with Rituximab.

## Discussion

IgG4-related disease (IgG4-RD) is a chronic systemic autoimmune inflammatory disease typically affecting older persons in the age range of 50 to 70 years. Global data on its incidence is limited and prevalence is estimated at 0.82 per 100 persons between 2007 and 2016. This might be underreported given the poor understanding and recognition of this disease [1,2].

It is typically characterized by extensive organ infiltration with lymphoplasmacytic cells, presence of storiform fibrosis, and obliterative phlebitis. IgG4- RD remains a diagnostic challenge as it is frequently misdiagnosed as malignancies especially due to its mass-forming presentation and multi-organ involvement [3].

The gold standard for the diagnosis of IgG4-RD remains characteristic histopathological findings on tissue biopsy. Ancillary evaluation including clinical, laboratory and imaging evaluations could be helpful especially in the setting of a high index of suspicion. Serum IgG4 levels are typically >135mg/dl , however, this may or may not correlate with the presence of disease and as such is not a required criteria for diagnosis. Albeit, elevated serum IgG levels tend to correspond with the extent of disease [2,3].

Commonly affected organs include the salivary glands, lacrimal glands, lungs, thyroid, lymph nodes, central nervous system, pituitary body, aorta, pancreas, liver, gallbladder, bile ducts, retroperitoneum, kidney, and prostate [4].

In its severe form, significant symptoms may arise in the form of obstruction or compression of surrounding organs due to tumor effect. It is important to consider IgG4-related disease when evaluating neoplastic conditions [5].

IgG4-related respiratory disease (IgG4-RRD)

IgG4-RRD may be detected incidentally. Patients with lung involvement could be asymptomatic or present with vague and non-specific symptoms like dyspnea, cough, chest discomfort. Non-constitutional symptoms have also been reported [6]. Lung involvement may present as organizing pneumonia, interstitial pneumonitis, organizing pneumonia, lymphomatosis granulomatosis, or solitary lung lesions. The pleura, airways, mediastinum, lung parenchyma, and adjacent vasculature may also be affected [6,7].

Most pulmonary imaging done in the setting of IgG4 related disease have reported varying pathologic patterns that include solid nodular density, ground-glass opacities, bronchiectasis, honeycombing, and bronchovesicular thickening [6,8].

Pleomorphic chest radiographic features in an individual patient should heighten suspicion for IgG-related disease. Lung biopsy may be required in some cases to make a definitive diagnosis [7,9]. This was unnecessary in our patient as the lesions responded well to treatment and remission was confirmed upon repeat imaging and decreasing IgG levels.

IgG4-related kidney disease (IgG4-RKD)

IgG-RKD is the most common prevalent urologic manifestation of IgG4-RD, often present as tubulointerstitial nephritis (TIN) and less often as membranous glomerulonephritis (MGN). Solitary renal mass, though rare, has also been described [9-11]. About 75% of patients presenting IgG4-RKD have extrarenal involvement [12]. The diagnostic criteria for IgG4-RKD proposed in 2011 by the Japanese society of nephrology are shown in (Table 1).

**Table 1:** 2011 Diagnostic criteria for IgG4-related kidney disease (IgG4-RKD) proposed by the Japanese Society of Nephrology Source: [13]

(1) Presence of some kidney damage, as manifested by abnormal urinalysis or urine marker(s) or decreased kidney function with either elevated serum IgG level, hypocomplementemia, or elevated serum IgE level
(2) Abnormal renal radiologic findings:
(a) Multiple low-density lesions on enhanced computed tomography
(b) Diffuse kidney enlargement
(c) Hypovascular solitary mass in the kidney
(d) Hypertrophic lesion of renal pelvic wall without irregularity of the renal pelvic surface
(3) Elevated serum IgG4 level (IgG4 ≥ 135 mg/dl)
(4) Histologic findings in the kidney
(a) Dense lymphoplasmacytic infiltration with infiltrating IgG4-positive plasma cells > 10/high-power field (HPF) and/or IgG4/IgG-positive plasma cells > 40%
(b) Characteristics fibrosis surrounding nests of lymphocytes and/or plasma cells
(5) Histologic findings in extrarenal organ(s):
Dense lymphoplasmacytic infiltration with infiltrating IgG4-positive plasma cells > 10/HPF and/or IgG4/IgG-positive plasma cells > 40% in extrarenal organ(s)
Definite:
(1) + (3) + (4) (a), (b)
(2) + (3) + (4) (a), (b)
(2) + (3) + (5)
(1) + (3) + (4) (a) + (5)
Probable:
(1) + (4) (a), (b)
(2) + (4) (a), (b)
(2) + (5)
(3) + (4) (a), (b)
Possible:
(1) + (3)
(2) + (3)
(1) + (4) (a)
(2) + (4) (a)
Appendix:
(1) Clinically and histologically, the following diseases should be excluded: Wegener’s granulomatosis, Churg-Strauss syndrome, and extramedullary plasmacytoma
(2) Radiologically, the following diseases should be excluded: malignant lymphoma, urinary tract carcinomas, renal infarction, and pyelonephritis (rarely, Wegener’s granulomatosis, sarcoidosis, and metastatic carcinoma)
(3) Cases with suspected disease according to the diagnostic algorithm are classified into probable or possible IgG4-RKD according to these criteria

Our patient presented with elevated IgG4 serum levels. His IgG4/IgG ratio was >40%, therefore meeting the required diagnostic threshold for IgG4-RD. Differential diagnoses like malignant disease, lupus erythematosus, Wegener’s granulomatosis, sarcoidosis, and metastatic carcinoma should be satisfactorily excluded [13,14]. Furthermore, there should be evidence of optimal response to glucocorticoid therapy. An elaborate classification criterion was developed in 2019 by the American College of Rheumatology and the European League Against Rheumatism (ACR/EULAR). It adopted a three-tier approach where an eventual composite score of 20 and above is consistent with a diagnosis of IgG4 -RD [15].

Recent studies now propose a better understanding of the pathology of a once poorly understood IgG-4 RD. Studies describing oligoclonal cellular expansion of B cells and CD4+ Cells, especially the cytotoxic subsets, is believed to play a central role in disease pathogenesis. The T follicular helper cells (IL-4 secreting subset of CD4+ cells) working in tandem with Interleukin-10 is thought to drive B cell maturation, resulting in IgG-4 class switching. The part played by macrophages in the production of fibrosis through its interplay with the acquired immune system and fibroblasts is currently being studied and provides potential favorable therapeutic advancements [16].

Treatment

The current recommendation for the initial treatment of IgG4 related disease are systemic corticosteroids [17]. There are no randomized control trials addressing standardized steroid dose regimen. However, a dose of 30 mg or higher daily for 1-2 weeks with steroid taper is used. There is a paucity of data supporting the use of rituximab in the event of steroid resistance or relapse. However, our patient failed corticosteroid therapy, but had a good response on IV Rituximab. Other immunosuppressive agents like methotrexate, mycophenolate mofetil, cyclophosphamide, and tacrolimus have also been used [10]. Surgery may be performed depending on the clinical manifestations. The risk of eventual relapse, despite initial response to systemic glucocorticoid therapy frequently occurs. Elevated IgG4 serum concentration, as well as extensive organ involvement, have been linked to an increased likelihood of relapse [18,19].

## Conclusions

IgG-RD is a chronic inflammatory disease that often runs an indolent course and may affect virtually any organ of the body. It could be misdiagnosed clinically as malignancy. Diagnosis remains a challenge. However, the recent EULAR diagnostic criteria are very comprehensive and would serve as a useful guide. The first line of treatment is systemic glucocorticoids. Rituximab may be used as a second line agent if there is intolerance or suboptimal response to steroids. Surgery may also be considered in cases where symptoms are related to mass effect from a pseudotumor.

## References

[1] Uchida K, Masamune A, Shimosegawa T, Okazaki K (2012). Prevalence of IgG4-related disease in Japan based on Nationwide Survey in 2009. Int J Rheumatol.

[2] Lang D, Zwerina J, Pieringer H (2016). IgG4-related disease: current challenges and future prospects. Ther Clin Risk Manag.

[3] Weindorf SC, Frederiksen JK (2017). IgG4-related disease: a reminder for practicing pathologists. Arch Pathol Lab Med.

[4] Stone JH, Zen Y, Deshpande V (2012). IgG4-related disease. N Engl J Med.

[5] Floreani A, Okazaki K, Uchida K, Gershwin ME (2021). IgG4-related disease: changing epidemiology and new thoughts on a multisystem disease. J Transl Autoimmun.

[6] Kobayashi H, Shimokawaji T, Kanoh S, Motoyoshi K, Aida S (2007). IgG4-positive pulmonary disease. J Thorac Imaging.

[7] Campbell SN, Rubio E, Loschner AL (2014). Clinical review of pulmonary manifestations of IgG4-related disease. Ann Am Thorac Soc.

[8] Xie LJ, Li JF, Liu Z (2017). Immunoglobulin G4-related lung disease presenting as lung cavitating mass and mimicking lung cancer. Arch Rheumatol.

[9] Okubo T, Oyamada Y, Kawada M, Kawarada Y, Kitashiro S, Okushiba S (2017). Immunoglobulin G4-related disease presenting as a pulmonary nodule with an irregular margin. Respirol Case Rep.

[10] Brito-Zerón P, Bosch X, Ramos-Casals M, Stone JH (2016). IgG4-related disease: advances in the diagnosis and treatment. Best Pract Res Clin Rheumatol.

[11] Bianchi D (2016). IgG4-related disease: what urologists should know. Int Urol Nephrol.

[12] Raissian Y, Nasr SH, Larsen CP (2011). Diagnosis of IgG4-related tubulointerstitial nephritis. J Am Soc Nephrol.

[13] Kawano M, Saeki T, Nakashima H (2011). Proposal for diagnostic criteria for IgG4-related kidney disease. Clin Exp Nephrol.

[14] Khosroshahi A, Carruthers MN, Stone JH, Shinagare S, Sainani N, Hasserjian RP, Deshpande V (2013). Rethinking Ormond's disease: "idiopathic" retroperitoneal fibrosis in the era of IgG4-related disease. Medicine (Baltimore).

[15] Wallace ZS, Naden RP, Chari S (2020). The 2019 American College of Rheumatology/European League Against Rheumatism classification criteria for IgG4-related disease. Ann Rheum Dis.

[16] Chen Y, Lin W, Yang H (2018). Aberrant expansion and function of follicular helper T cell subsets in IgG4-related disease. Arthritis Rheumatol.

[17] Carruthers MN, Topazian MD, Khosroshahi A (2015). Rituximab for IgG4-related disease: a prospective, open-label trial. Ann Rheum Dis.

[18] Grewal K, Cohen P, Kwon JS, Kaufman DA (2016). IgG4-related disease presenting as a lung mass and weight loss: Case report and review of the literature. Respir Med Case Rep.

[19] Campochiaro C, Della-Torre E, Lanzillotta M (2020). Long-term efficacy of maintenance therapy with Rituximab for IgG4-related disease. Eur J Intern Med.

